# Rethinking Tourist Wellbeing through the Concept of Slow Adventure

**DOI:** 10.3390/sports7080190

**Published:** 2019-08-08

**Authors:** Jelena Farkić, Steve Taylor

**Affiliations:** Centre for Recreation and Tourism Research, West Highland College UHI, Carmichael Way, Fort William PH33 6FF, UK

**Keywords:** slow adventure, time, slowness, wellbeing

## Abstract

The necessity for humans inhabiting the 21st century to slow down and take time to carry out daily practices frames the discourse of this research note. We suggest reconceptualising tourist wellbeing through the concept of slow adventure, as a response to the cult of speed and as a vehicle for engaging in deep, immersive and more meaningful experiences during journeys in the outdoors. We suggest that slow adventure has the potential to improve people’s general health and wellbeing through mindful enjoyment and consumption of the outdoor experience and thus bring people back to a state of mental and physical equilibrium. In so doing, we argue that extending the concept to include discussions around the psychological and social aspects of slow adventure is needed.

## 1. Introduction

This commentary is set within the discourse that draws attention to the necessity for slowing down and taking time to carry out practices in life in order to construct richer, deeper and more meaningful experiences. The argument is taken further to suggest that slow adventure, a tourism concept, inspired by the global slow movement and *friluftsliv* (the Nordic philosophy of outdoor life), may be an antidote to the societal condition characterised by prevalent psychological illbeing. While there is a large corpus of research discussing health, wellbeing, outdoor activities, adventure or positive psychological effects of tourism in general, studies that specifically interrogate wellbeing in the context of adventure tourism are limited. We therefore point to the necessity of continued research into potentials of slow adventure, as a way of ‘being’ on holiday and consuming the outdoors at a slower pace, to improve the health and wellbeing of humans inhabiting the 21st century.

People’s ways of life have drastically changed in highly developed societies. Stress-related and mental health problems are an ever-growing concern. The World Health Organisation envisaged that in 2020 depression and related illnesses will become the largest cause of ill-health [1]. It is no surprise that in an era of prevalent risks, insecurities and anxieties, individuals increasingly search for ontological security in all aspects of their lives [2,3,4]. People seem ‘trapped’ within their own delirious discourse and within the structures of the digitalised, urbanised and industrialised world that seem to have been only designed to confine us. It is claimed that time deprivation is one of the consequences of high-speed societies [5,6]. Therefore, as a response to the accelerated tempo of living, slowness has been gradually introduced to various spheres of people’s lives. It has been incorporated into concepts such as food, cities or travel, to denote the value of time invested in the consumption, appreciation and delayed, rather than instant, gratification of experiences. Slowness has a special focus on learning how to value and cultivate the sense of time and “restore meaning, authenticity, security or identity to time-deprived subjects” [7] (p. 369). Likewise, slow travel celebrates simple, local, traditional, sensory and affective aspects of the experiences generated through movements at a slower pace and immersion in the destination and local way of life. It explains one’s desire to connect to pleasant, enjoyable and meaningful things, while at the same time disconnecting from the stressful and disturbing stimuli with which the external world is overly saturated [8,9]. 

## 2. Health Benefits of Nature-Based Tourism

It is claimed that people’s quality of life may be greatly improved through tourism [10,11,12]. As a ‘social force’ [13] it can in many ways create comfortable, hospitable spaces in which people can temporarily slow down, feel safe and secure, dwell, bond and belong, and in which they can, in the basic, primitive, ontological sense, feel well. Thus, vacations have been increasingly ‘prescribed’ [14]. Ever more people are travelling for the purpose of improving their general health and wellbeing, which has prompted redesigning and developing diverse tourism products that promote both physical and mental health. This espouses preventative rather than curative approaches to personal health management and the visitation of destinations and facilities that support this. Many destinations worldwide are now creating their products to meet the needs of postmodern consumers interested in lifestyle-based wellness and overall wellbeing [15]. In particular, short nature-breaks have the power to ‘fix’ the body, spirit and mind, and offer compensation for people’s alienated, fast-paced lives [16]. These therapeutic and restorative powers of nature have been highlighted [17,18]. Various authors raise awareness of the necessity for continued research into the benefits of tourism on people’s wellbeing. By way of example, the interplay of tourism, health, wellbeing and protected areas has been recently discussed [19], illuminating the ways in which these four broad fields of research co-exist. Simultaneously looking at the preservation of protected areas on the one hand, and the health and wellbeing of their consumers on the other, the authors aim to deepen our understanding of the synergy of these themes and suggest ways in which together they can bring long-term benefits for multiple agents participating in this process. There have been correlations between spending time outdoors and people’s subjective wellbeing, which is directly linked to people’s overall happiness [20] and anxiety reduction [21]. The concept of wilderness therapy [22] has also brought some in-depth insights into the health benefits of undertaking activities in natural environments. 

Therefore, either being or being active in nature is claimed to have noteworthy physical, mental, emotional and spiritual effects on people’s physical and mental health [10,23,24,25,26]. In reconnecting with the natural world, some individuals opt for travelling to unfamiliar and remote wild places, releasing their ‘adventurous’ spirit and searching for their ‘authentic’ being. Adventure, however, has been mostly associated with a purchasable short-term holiday experience and as a marketing hook for potential consumers who wish to engage in what Varley et al. [27] termed as ‘scream-n-go’ experiences. Adventure tourism, through commodification of such experiences, has become one of the fastest growing niche tourism forms, being researched from multiple perspectives to date. Rantala et al., however, stated that adventure tourism is a “concept which has different meanings and uses depending on context”, which is “too broad and too fluid” [28] (p. 9). Commercially, it is defined as a product “where the principal attraction is an outdoor activity that relies on features of the natural terrain, generally requires specialised equipment, and is exciting for the tour clients” [29] (p. 2). It mostly promotes ‘hard’ or fast adventure activities such as climbing, mountaineering, white-water kayaking, bungee jumping or snowmobiling, all of which are usually embedded into the natural environment.

## 3. Slowing Down, Immersion and Adventure

However, there is more to adventure tourism and outdoor activities. Apart from the ‘classic’ outdoor adventure sports, tourists are offered ‘softer’, more immersive activities in order to experience, know and feel cultural landscapes. These experiences are also claimed to be highly embodied, with an emphasis on the extension of time, comfort and deep multisensorial appreciation of and engagement with the surrounding places and social groups [23]. This is where the materialisation of ‘slow adventure’ becomes important. In conceptualising slow adventure, Varley and Semple [16] distilled its four critical elements: time, nature, passage and comfort. Time embodies itself in awareness of its passing during the outdoor journeys; nature refers to the natural setting and access to it; passage, both physical and spiritual, is the navigation through time, space and the self; comfort implies being at ease with the unusual challenges throughout the journey, or reconnection with the place and the self. Evidently, the concept is juxtaposed to the adrenaline-pumping outdoor adventure activities and extreme sports used to traditionally define adventure tourism. 

However, the temporal aspect of the tourism experience and the commodification of slowness seem crucial here. The authors explain that slow journeys “unfold at human pace, meals take time to prepare; time is spent directly in the effort of journeying and living” [16] (p. 86). Consequently, the slow pace of doing things allows for the unravelling of other qualities that are germane to the slow adventure concept. For example, spending extended time in nature, and physical activity combined with relaxation of the mind and intellectual stimulus, allows for subjective comfort, the fitness of body and wellness of mind and spirit. This may be achieved through collectively partaking in recreational activities, such as kayak or canoe expeditions, multi-day treks, cooking foraged foods, pitching a tent in a forest or telling stories around a crackling campfire. In so doing, people are given the opportunity to immerse themselves into prolonged interactions between self and the world. Such activities are usually undertaken in small groups, and although safer and less risky, they require the presence of an expert, a skilled guide, to interpret, mediate and navigate people through unfamiliar wild spaces, negotiate harsh environments and make such experiences more available even for less skilled participants [27,30]. Such experiences facilitate the generation of social capital and the creation of deep, memorable experiences. Ultimately, slow adventure may in many ways be compared to the Danish ‘hygge’, explained as “a state of pleasant wellbeing and security, with a relaxed frame of mind and open enjoyment of the immediate situation in all its small pleasures” [31] (p. 54) cf. [27].

Recently, psychological and social wellbeing benefits of outdoor adventure tourism have gained increased attention among researchers [32,33]. Filep et al. [34], for example, tackled the issue of wellbeing in this context and pointed to the absence of more substantial research surrounding this topic. To date, there have been few studies granting attention to the relations between wellbeing and adventure tourism practices, and these have tended to adopt a more generic approach, which underplays the inherent value of slow, immersive ‘being’ in nature, rather than actively ‘doing’. For example, Kulczycki and Lück’s study suggested that “various components of the adventure tourism experience, such as place attachment, calculated risk taking, achievement and accomplishment, social interaction, have the capability to actively contribute not only to health, but also to wellbeing” [35] (p. 176). Similarly, in conceptualising adventurous nature sport (ANS) in relation to wellbeing, Houge Mackenzie and Brymer [36] framed their study within positive psychology and discussed the eudaimonic and hedonic outcomes of undertaking ANSs. The authors claim that such activities enhance physical health and psychological wellbeing in a number of ways, for example building resilience and fulfilling psychological needs, thus calling for further research into social aspects of ANSs in relation to wellbeing and flourishing. 

## 4. Conclusions

In general, adventure tourism has the potential to address current societal conditions and offer possibilities for re-establishing people’s connection with both natural and social environments. Building on the postulates of slowness, the aim of slow adventure is to introduce consumers to an alternative dimension of adventure and the simplicity of just *being* in the outdoors. It can partly aid the treatment of ‘affluenza’ and move people away from their languishing towards flourishing [37]. Therefore, this commentary suggests that adventure tourism researchers have much to gain from further explorations of the health and wellbeing benefits of undertaking slow activities on holidays. Through their highly embodied activities, immersive research should investigate how people may successfully recreate familiar, comfortable spaces in which they experience longed-for feelings of reconnection, restoration, reunion, regeneration or recreation, and make a meaningful contribution to their improved wellbeing. Crucial here is to extend the research into temporal, psychological and social dimensions of the slow adventure concept as a commodified tourism product. In particular, psychological, sociological, philosophical and ecosophical concepts, such as spirituality, mindfulness, friluftsliv, flow, sense of place, communitas, ontological security, dwelling or aesthetic experiences, may provide theoretical avenues in future explorations that explore how ‘slowness’ can conduce the wellbeing of fast-living and fast-moving inhabitants of the 21st century. 
